# Further Validating the Robotic Microsurgery Platform through Preclinical Studies on Rat Femoral Artery and Vein

**DOI:** 10.1055/a-2460-4940

**Published:** 2025-01-17

**Authors:** Jeongmok Cho, Donggeon Kim, Taehyun Kim, Changsik John Pak, Hyunsuk Peter Suh, Joon Pio Hong

**Affiliations:** 1 Department of Plastic and Reconstructive Surgery, University of Ulsan, College of Medicine, Seoul Asan Medical Center, Seoul, Korea

**Keywords:** robotic microsurgery, supermicrosurgery, robotic-assisted surgery

## Abstract

**Background:**

This research aims to validate the proficiency and accuracy of the robotic microsurgery platform using rat femoral vessel model.

**Methods:**

A total of 256 rat femoral vessels were performed, half using robotic and the other by manual microanastomosis by eight microsurgeons with less than 5 years of experience given eight trials (rats) each. Vessel demographics, proficiency (duration of suture and Structured Assessment of Robotic Microsurgical Skills [SARMS]), and accuracy (patency and scanning electron microscopic [SEM]) were analyzed between the two groups.

**Results:**

Using the robot, an average of four trials was needed to reach a plateau in total anastomosis time and patency. Significant more time was required for each vessel anastomosis (34.33 vs. 21.63 minutes on the eighth trial, *p* < 0.001) one factor being a higher number of sutures compared with the handsewn group (artery: 7.86 ± 0.51 vs. 5.86 ± 0.67, *p* = 0.035, vein: 12.63 ± 0.49 vs. 9.57 ± 0.99, *p* = 0.055). The SARMS scores became nonsignificant between the two groups on the fourth trial. The SEM showed a higher tendency of unevenly spaced sutures, infolding, and tears in the vessel wall for the handsewn group.

**Conclusion:**

Using the robot, similar patency, accuracy, and proficiency can be reached through a fast but steep learning process within four trials (anastomosis of eight vessels) as the handsewn group. The robotic anastomosis may take longer time, but this is due to the increased number of sutures reflecting higher precision and accuracy. Further insight of precision and accuracy was found through the SEM demonstrating the possibility of the robot to prevent unexpected and unwanted complications.

Since the pioneering work of Dr Buncke, microsurgery not only has expanded the surgeon's ability to reconstruct but has evolved into new principles and strategies. 1 2 3 4 Once considered the highest rung of the reconstructive ladder, success rate of over 95 to 97% is commonly seen. 1 5 6 7 Nevertheless, there are 3 to 5% of flap failures, which are often unclear on why it occurred. 8

With the recent introduction of supermicrosurgery concept, it now allows to expand the spectrum of reconstruction while being minimal invasive and efficient. 1 4 9 10 11 12 13 Furthermore, once thought untreatable, lymphedema surgery using supermicrosurgery concepts is now a reality. 1 10 However, to obtain the skills for supermicrosurgery requires long hours of training and constant exposure to cases that allows surgeons to develop skills such as stability, dexterity, and motion precision. 14 15 Thus, despite the known benefits, it is still not readily available in many parts of the world.

Today, robotics has become the gold standard for various surgical specialties allowing surgeons to perform delicate and complex procedures that may be difficult or impossible with other methods. 16 17 Often, robotic surgery makes minimally invasive surgery possible with their capability to reduce tremor and increase scaling potential resulting in fewer complications, reduced admission time, faster recovery, less scars, and infection. 18 Thus, the same idea and principle may apply to a finer and more delicate microsurgery and supermicrosurgery. 19 20 21 22 The robotic platforms are now being clinically introduced and users have reported successful application for free flap surgery and lymphedema surgery with precision. 21 23 24 25 26 27 28 29 However, there are still many questions to be answered in regard to the application, efficiency, learning curve, telemedicine, artificial intelligence, and others.

The purpose of this preclinical study was to assess the proficiency and accuracy of the robotic microsurgical system and conduct an in-depth examination of the learning curve for microsurgeons. Our aim is to affirm the benefits of robotic assistance and determine the future direction for advancement.

## Methods

### Experimental Design

A total of 64 Lewis rats were used in the experimental protocol approved by the Animal Care and Use Committee of University of Ulsan, College of Medicine, Asan Medical Center. The anastomosis was performed bilaterally on both femoral artery and vein. One side used the robot, whereas the contralateral underwent conventional handsewn microanastomosis. Once the vessel dissection was made and prepared for transection and anastomosis, the diameters of both veins and arteries were measured. The entire microanastomosis procedure was video-recorded for evaluation. After completion, patency was confirmed after 0 and 30 minutes, and total number of stitches was documented. The vessel containing the microanastomosis was collected after the eighth trial to obtain histology and scanning electron microscope (SEM). The overall evaluation was made by comparing the robot-sewn versus the handsewn group.

### Robotic Platform for Microsurgery

**Fig. 1:**
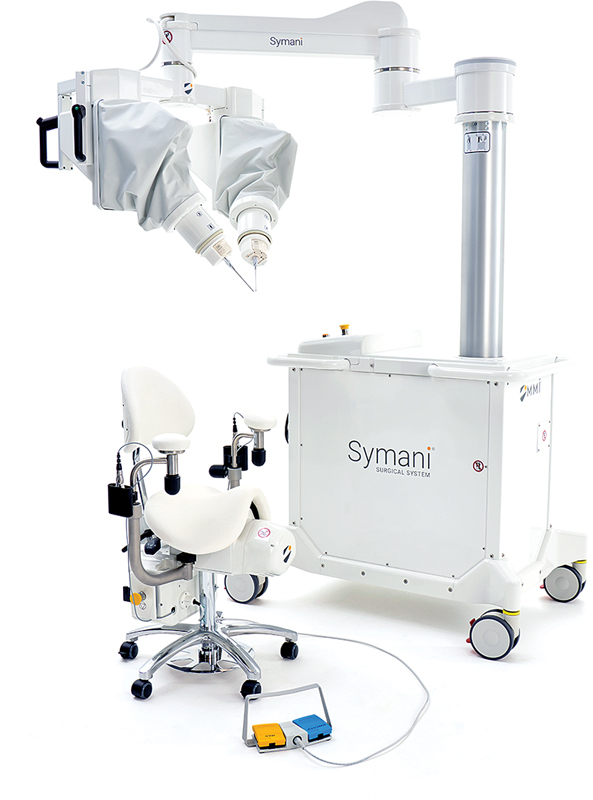
The Symani surgical system, which provides robotic arms for microsurgery.

The Symani surgical system (MMI [Medical Microinstruments], Pisa, Italy) employs teleoperation principles, scaling down the surgeon's hand motions by 7 to 20 folds while eliminating physiological hand tremors. The robotic arm and wrist move freely without a limited range of motion guaranteeing both dexterity and precision. With a long pincer-like manipulator and a footswitch from the master console, the surgeon can replicate movements from the manual instruments. There are two types of robot arms, one with side with a needle holder embedded with scissors to cut sutures and the other mimicking the jeweler forceps ( Fig. 1 ).

### Surgeon Selection

Eight microsurgeons with less than 5-year experience were enrolled after a session of training program (dry-run) acquiring proficiency in safety and utilization before performing the experiment.

### Anastomosis Skill and Quality Evaluation

**Supplementary Video S1** Elaboration of each segment of the suturing cycle.


In addition to the direct evaluation of anastomosis such as time and number of stitches required, proficiency was evaluated for skill, and accuracy was evaluated for the quality of the anastomosis.

1. Proficiency validation
  - Total and segmented duration of anastomosis
With the recorded video, time was measured for the overall duration (in seconds). The recording was further broken down to evaluate a single suturing cycle and divided into four segments. First, time to pick up the needle. Second, the next phase till the needle starts to penetrate the wall. Third, the next phase till the end of the suture with a completed knot. Fourth, time required to cut the suture. During the experiment, two independent evaluators assessed the video independently ( Supplementary Video S1 , available in online version only).
  - Structured Assessment of Robotic Microsurgical Skills
The skills for robotic microsurgery encompass a blend of conventional microsurgical principles along with additional adaptation to utilization of microsurgery robotic arms. 30 It can be systematically evaluated using the Structured Assessment of Robotic Microsurgical Skills (SARMS tool; biological dexterity, tissue handling, microsuture placement, knotting technique, and motion with a scale from 0 to 5 points. The overall performance was evaluated based on a perfect score of 10) presented and validated by the MD Anderson group. 31 The changes in candidates' skills were recorded with each trial for both robotic and handsewn techniques. Two independent evaluators evaluated the video taken during the experiment.
2. Accuracy validation
  - Patency test
The restoration of vascular flow after patency completion test (milking test) is directly tied to the quality and precision of the anastomosis performance. 32 Patency evaluation was conducted at 0 and 30 minutes after anastomosis for both veins and arteries. In addition to positive milking test, leakage was evaluated. The criteria for defining the condition of the anastomosis after 30 minutes were as follows: “no leakage;” some leakage initially but it naturally stopped, “partial leakage;” leakage requiring additional stitches, “severe leakage.”
  - Scanning electron microscopic analysis
    After the eighth trial, specimen was collected for the SEM analysis to evaluate the morphology of the microsuture, corresponding to the quality of the wall-to-wall coaptation. Under the SEM, the quality and distance between each knot, foreign body on the sutures, injured backwall, infolding of vessel wall, tearing of the vessels wall were clearly observed.

### Statistical Analysis

The independent *t* -test was used to compare the overall and segmented anastomosis duration differences between the two groups. To verify the differences in proficiency across difference time points, repeated measures analysis of variance was performed. The chi-square test was used to compare the vessel demographics. The data underwent analysis using IBM SPSS version 21.0 for Windows (IBM Corp., Armonk, NY) and GraphPad Prism Version 5.0. The *p* -value < 0.05 was considered statistically significant.

## Results

### Vessel Demographics and Anastomosis Evaluation

Each microsurgeon performed total of 32 microasnastomosis (16 femoral arteries and 16 veins), half using the robot and the other half handsewn. A total of 256 microanastomosis from eight microsurgeons were evaluated and evaluated under two groups: robot-sewn and handsewn. The diameters of both femoral arteries and veins showed no significant difference in the diameters used for both Symani-sewn and handsewn groups (arteries: 0.65 ± 0.04 mm vs. 0.55 ± 0.05 mm, *p* = 0.150 and veins: 0.99 ± 0.03 mm vs. 0.98 ± 0.07, *p* = 0.852). Upon completion of anastomosis, the total number of arterial stitches performed were significantly higher for the robot-sewn group (7.86 ± 0.51 vs. 5.86 ± 0.67, *p* = 0.035). The total number of venous stitches performed were significantly higher for the robot group (12.63 ± 2.65 vs. 9.57 ± 2.54, *p* = 0.045; Table 1 ).

**Table 1:** Demographics of arteries and veins and the evaluation of stitches required for a single vessel anastomosis

		Symani	Hand	*p* -Value a
Diameter (mm)	Artery	0.65 ± 0.27	0.55 ± 0.25	0.15
	Vein	0.99 ± 0.03	0.98 ± 0.07	0.148
Number of stitches	Artery	7.86 ± 2.01	5.86 ± 2.22	0.035
	Vein	12.63 ± 2.65	9.57 ± 2.54	0.045

a Independent *t* -test was performed for statistical significance.

### Duration of Anastomosis: Total and Segmented Time Evaluation

When analyzing the total duration of a single vessel anastomosis, the initial trial using robotic platform was significantly longer (3,674 ± 23.69 seconds [61.23 minutes] vs. 2,081 ± 20.83 seconds [34.68 minutes]). By the last eighth trial, although reduced in total anastomosis time, the robot still took significantly longer (2,060 ± 29.11 seconds [34.33 minutes] vs. 1,298 ± 28.65 seconds [21.63 minutes]; Table 2 ).

**Table 2:** Analysis of total duration for single vessel anastomosis for each trial and the tendency of change in duration over the trials

	Time (s) mean ± SD		*p* -Value a
	Symani	Hand	
1st	3,674 ± 23.69	2,081 ± 20.83	<0.001
2nd	2,783 ± 16.38	1,332 ± 37.50	<0.001
3rd	2,100 ± 21.52	1,413 ± 22.53	<0.001
4th	2,333 ± 27.91	1,358 ± 22.13	<0.001
5th	2,088 ± 13.89	1,320 ± 14.33	<0.001
6th	2,073 ± 35.72	1,285 ± 14.28	<0.001
7th	2,094 ± 20.11	1,310 ± 34.86	<0.001
8th	2,060 ± 29.11	1,298 ± 28.65	<0.001
	*p* -Value b		
	Symani	Hand	Time*group
1st–4th	0.000	0.072	0.000
5th–8th	0.103	0.595	0.169

a Independent t-test was performed for comparison of average.

b Repeated measure analysis of variance, comparing Symani and hand suture group (total time).

**Fig. 2:**
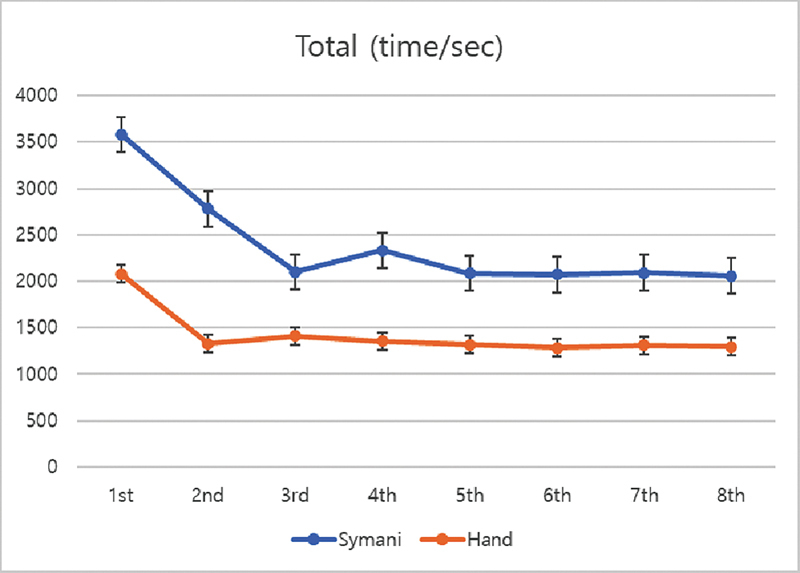
Total anastomosis time required for a single vessel over each trial. The total time is higher for the robot-sewn group. Note that the arteries plateaus from the third trial while the vein plateaus from the fifth trial.

In the analysis conducted to compare duration over each trial (trend), the duration for each anastomosis from the first to the fourth trial was significantly reduced for the Symani-sewn group ( *p* < 0.05) and from the fifth to the eighth trial without significant change ( *p* = 0.169), suggesting proficiency can be reached after the fourth trial. In the handsewn group, the suture time did not significantly decrease after the second trial. The overall trend remained similar between the two groups from the fifth trial ( Table 2 , Fig. 2 ).

**Fig. 3:**
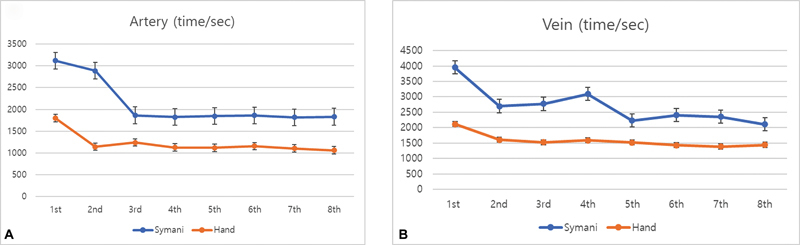
Anastomosis time required for artery and vein over each trial. The artery plateaus from the third trial when using the robot. The overall time for artery takes longer using the robot ( **A** ). The vein plateaus from the fifth trial when using the robot. The overall time for vein anastomosis takes longer using the robot ( **B** ).

When comparing arteries and veins separately, the reduction of artery anastomosis time was significant till the third trial and the trend from the fourth trial did not show any significant difference ( *p* = 0.059). For the veins, there were significant changes till the fifth trial, but no difference afterward ( Table 3 , Fig. 3 ). Thus the proficiency of the artery is achieved earlier than the vein.

**Table 3:** Analysis of total duration for single artery and vein anastomosis for each trial and the tendency of change in duration over the trials

**(a) Artery time comparison**			
	**Time (s) mean ± SD**		***p*** ** -Value a**
	**Symani**	**Hand**	
1st	3,118 ± 24.64	1,799 ± 22.80	<0.001
2nd	2,895 ± 42.22	1,145 ± 25.32	<0.001
3rd	1,865 ± 35.22	1,243 ± 17.32	<0.001
4th	1,827 ± 24.56	1,128 ± 16.16	<0.001
5th	1,848 ± 18.65	1,122 ± 14.89	<0.001
6th	1,864 ± 35.42	1,158 ± 17.65	<0.001
7th	1,821 ± 31.33	1,106 ± 16.35	<0.001
8th	1,833 ± 38.63	1,064 ± 12.33	<0.001
	***p*** ** -Value b**		
	**Symani**	**Hand**	time*group
1st–3rd	0.000	0.000	0.000
4th–8th	0.059	0.052	0.235
( **b) Vein time comparison**			
	**Time (s) mean ± SD**		***p*** **-value**
	**Symani**	**Hand**	
1st	3,956 ± 33.42	2,118 ± 25.22	<0.001
2nd	2,700 ± 21.28	1,607 ± 27.51	<0.001
3rd	2,772 ± 19.86	1,527 ± 18.84	<0.001
4th	3,092 ± 10.63	1,588 ± 23.32	<0.001
5th	2,233 ± 17.56	1,518 ± 14.28	<0.001
6th	2,358 ± 20.58	1,435 ± 12.89	<0.001
7th	2,355 ± 22.33	1,392 ± 13.33	<0.001
8th	2,205 ± 19.74	1,443 ± 17.17	<0.001
	***p*** ** -Value c**		
	**Symani**	**Hand**	time*group
1st–5th	0.000	0.000	0.000
6th–8th	0.282	0.071	0.581

a ndependent t-test was performed for comparison of average.

b Repeated measure analysis of variance, comparing Symani and hand suture group (artery time).

c Repeated measure analysis of variance, comparing Symani and hand suture group (vein time).

**Fig. 4:**
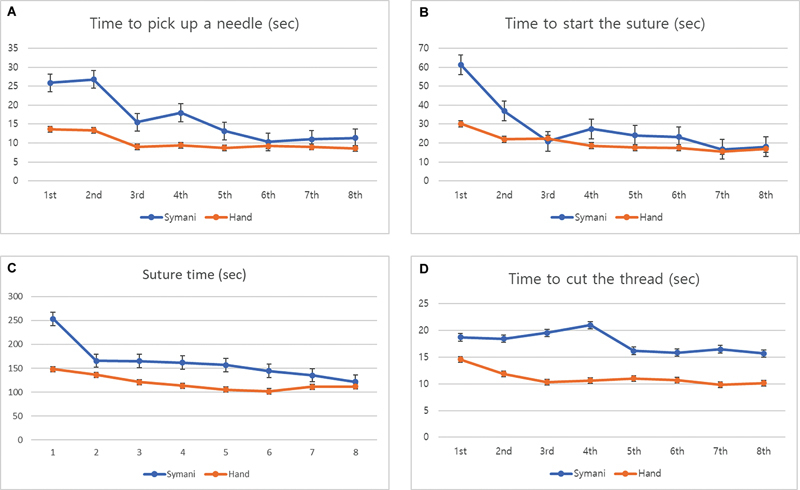
When analyzing each segment of a single suturing cycle, the time needed to pick up the needle became similar from the sixth trial ( **A** ) and the time needed to start the suture after picking up the suture was similar from the third trial ( **B** ) between the robot and handsewn groups. The time required to complete a single suture from the point of needle penetration showed a gap but became relatively constant from the second trial but reached almost the same time on the eighth trial ( **C** ). The final segment needing time to cut the suture after tying the know reached a constant gap on the fifth trial ( **D** ).

When analyzing each segment of a single suturing cycle, the time needed to pick up the needle became similar from the sixth trial with 10.33 ± 1.21 and 9.21 ± 1.15 seconds, respectively, for Symani and handsewn groups. The time needed to start the suture after picking up the suture was similar from the third trial with 20.93 ± 2.74 and 22.37 ± 4.73 seconds, respectively. The time required to complete a single suture from the point of needle penetration showed a gap and on the eighth trial reaches similar time with 121.33 ± 34.11 and 111.67 ± 32.50 seconds, respectively. The final segment needing time to cut the suture after tying the knot reached a constant gap from the fifth trial taking longer time using the robot (16.17 ± 1.35 vs. 11.01 ± 2.11 seconds; Table 4 , Fig. 4 ).

**Table 4:** Evaluation of the anastomosis time based on each phase (in seconds)

		1st	2nd	3rd	4th	5th	6th	7th	8th
1st phase Pick up a needle	Symani	25.92 ± 3.82	26.78 ± 3.89	15.53 ± 2.40	18.00 ± 2.24	13.17 ± 2.35	10.33 ± 1.21	11.00 ± 2.45	11.35 ± 2.11
	Hand	13.69 ± 1.90	13.40 ± 2.85	9.00 ± 0.98	9.43 ± 1.21	8.75 ± 1.37	9.21 ± 1.15	8.94 ± 1.64	8.59 ± 1.40
2nd phase Start the suture	Symani	61.38 ± 7.17	36.91 ± 6.32	20.93 ± 2.74	27.43 ± 2.55	24.00 ± 1.53	23.17 ± 1.35	16.67 ± 1.85	17.99 ± 1.25
	Hand	30.15 ± 4.98	21.93 ± 4.00	22.37 ± 4.73	18.64 ± 1.99	17.66 ± 2.41	17.45 ± 2.85	15.55 ± 1.51	16.88 ± 1.84
3rd phase Completion of suture	Symani	253.00 ± 71.31	165.61 ± 46.91	165.00 ± 35.36	161.77 ± 46.76	156.73 ± 36.95	144.09 ± 69.7	135.26 ± 65.2	121.33 ± 34.11
	Hand	147.95 ± 55.42	135.98 ± 37.02	120.74 ± 34.73	113.25 ± 32.58	105.35 ± 34.55	101.68 ± 64.21	109.23 ± 61.41	111.67 ± 32.50
4th phase Cutting the thread	Symani	18.70 ± 2.15	18.43 ± 1.90	19.53 ± 1.91	20.97 ± 1.76	16.17 ± 1.35	15.83 ± 1.58	16.46 ± 1.36	15.66 ± 1.24
	Hand	14.55 ± 2.43	11.86 ± 1.66	10.34 ± 1.06	10.63 ± 1.21	11.01 ± 2.11	10.67 ± 1.24	9.85 ± 1.43	10.12 ± 1.68

### Evaluation after Proficiency

After proficiency was reached, the anastomosis time and number of stitches were compared from the seventh trial onward. The number of stitches at the seventh and eighth trials were 10.44 ± 4.48 and 10.63 ± 4.41 being significantly higher for the robot-sewn group compared with 8.39 ± 4.21 and 8.13 ± 5.01 of the handsewn group ( *p* = 0.012, 0.048) showing more sutures are required using the robot. However, when evaluating the time per stitch, there was no statistical difference. On the seventh and eighth trials, the time per stitch were 135.26 ± 65.2 and 121.33 ± 34.11 seconds using the robot compared with 109.23 ± 61.41 and 111.67 ± 32.50 seconds for the handsewn group ( *p* = 0.22, 0.243; Table 5 ). Once proficiency is reached, the actual time per stitch becomes insignificant between the two groups.

**Table 5:** Evaluation of anastomosis time per stitch after proficiency is achieved at seventh and eighth trials

**(a) 7th trial**				
		**Symani**	**Hand**	***p*** ** -Value a**
Number of stitches	Total	10.44 ± 4.48	8.39 ± 4.21	0.012
	Artery	8.46 ± 5.36	6.15 ± 4.98	0.101
	Vein	12.42 ± 4.73	10.64 ± 4.95	0.045
Time per stitch (s)	Total	135.26 ± 65.2	109.23 ± 61.41	0.22
	Artery	111.78 ± 33.92	85.98 ± 31.58	0.061
	Vein	158.74 ± 41.22	132.44 ± 38.47	0.103
**(b) 8th trial**				
		**Symani**	**Hand**	***p*** ** -Value a**
Number of stitches	Total	10.63 ± 4.41	8.13 ± 5.01	0.048
	Artery	8.21 ± 3.91	6.01 ± 3.14	0.046
	Vein	13.06 ± 3.34	10.25 ± 3.19	0.029
Time per stitch (s)	Total	121.33 ± 34.11	111.67 ± 32.50	0.243
	Artery	100.45 ± 21.57	94.72 ± 19.13	0.065
	Vein	142.21 ± 26.47	128.62 ± 25.28	0.164

a Independent t-test was performed for statistical significance.

### Structured Assessment of Robotic Microsurgical Skills Scores

**Fig. 5:**
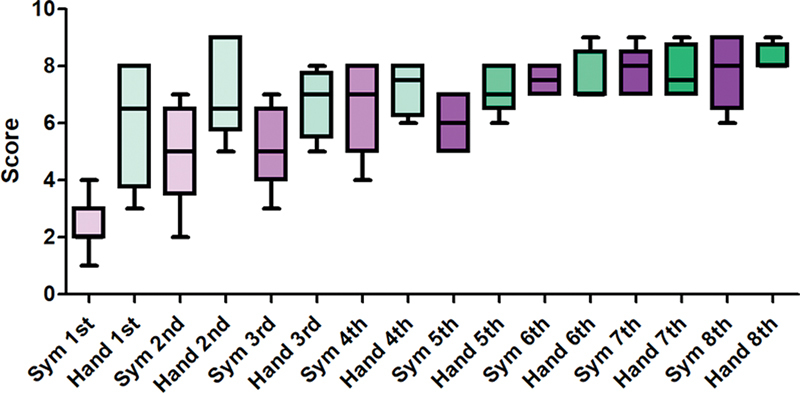
The SARMS score compared across each trial showed a steady increase in both groups, and from the fourth trial onward, there was no significant difference in scores. SARMS, Structured Assessment of Robotic Microsurgical Skills.

The SARMS score compared across each trials showed a steady increase in both groups. The first ( *p* = 0.036), second ( *p* = 0.008), and third ( *p* = 0.016) trials showed a statistically significant difference in the average scores between the two groups, but from the fourth trial onward, there was no significant difference in scores ( Tables 6 , 7 ; Fig. 5 ).

**Table 6:** The average of Structured Assessment of Robotic Microsurgical Skills score at each trial

**(a) Symani**								
	**1st**	**2nd**	**3rd**	**4th**	**5th**	**6th**	**7th**	**8th**
Score	2.44	4.78	5.2	6.6	6	7.5	7.8	7.8
Motion	1.33	2.78	2.6	3.4	3	3.7	3.8	3.8
Speed	1.11	2.22	3	3.4	3.5	4	4	3.8
Needle handling	2.22	2.89	3	3.6	4	3.5	4.1	4.2
Tissue handling	2.11	2.33	2.8	3.4	3	3	3.5	4
Suture placement	1.78	2.56	2.6	3.6	3.5	3.5	4	3.9
Knot technique	2.89	3.67	3.6	4.2	4.5	4.5	4.5	4.4
**(b) Hand**								
	**1st**	**2nd**	**3rd**	**4th**	**5th**	**6th**	**7th**	**8th**
Score	6	7	6.75	7.25	7.2	7.6	7.75	8.25
Motion	3.17	3.67	3.75	3.75	3.8	3.8	4	4
Speed	3.5	3.67	3.5	3.5	3.4	3.2	3.5	3.5
Needle handling	3.83	3.67	3.5	3.5	3.6	3.6	3.75	4
Tissue handling	3.33	3.33	3.75	3.75	3.6	3.8	4	4.5
Suture placement	3	3.17	3.25	3.5	3.6	3.6	3.75	3.75
Knot technique	3.67	4.33	4.25	4.5	4.2	4.2	4.25	4.25

**Table 7:** Comparison of total Structured Assessment of Robotic Microsurgical Skills score over each trial

	1st	2nd	3rd	4th	5th	6th	7th	8th
Symani	2.44 ± 0.29	4.75 ± 0.57	5.20 ± 0.66	6.60 ± 0.75	6.0 ± 0.58	7.5 ± 0.29	7.8 ± 0.37	7.8 ± 0.58
Hand	6.0 ± 0.86	7.0 ± 0.68	6.75 ± 0.63	7.25 ± 0.48	7.20 ± 0.48	7.60 ± 0.40	7.75 ± 0.48	8.25 ± 0.25
*p* -Value a	0.036 b	0.008 b	0.016 b	0.624	0.252	0.638	0.638	0.495

a Independent t-test was performed for comparison of average.

b Statistically significant.

### Patency Test and Leakage Evaluation

At 30-minute postmicroanastomosis, except for two vein thrombosis using the robot and two vein thrombosis by hand during the early trials, all others showed 100% patency.

**Fig. 6:**
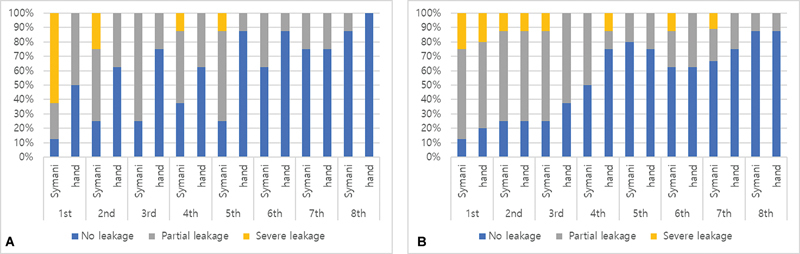
The incidence of leakage after the anastomosis is shown for arteries ( **A** ) and veins ( **B** ).

For arterial anastomosis, there was no severe leakage from the sixth trial. Anastomosis success rate without severe leakage was 37.5, 75.0, 100, 87.5, 87.5, 100, 100, 100%, respectively, from first to eighth trial with Symani robots. For the vein anastomosis, there was no severe leakage from the eighth trial and the success rate without severe leakage was 75.0, 87.5, 87.5, 100, 87.5, 87.5, 100, 100%, respectively, from the first till the eighth trial. There was no statistical difference in the number of leaking vessels in either artery ( *p* = 0.083) and vein ( *p* = 0.056) between the two groups ( Fig. 6 ).

### Scanning Electron Microscopic Analysis

**Fig. 7:**
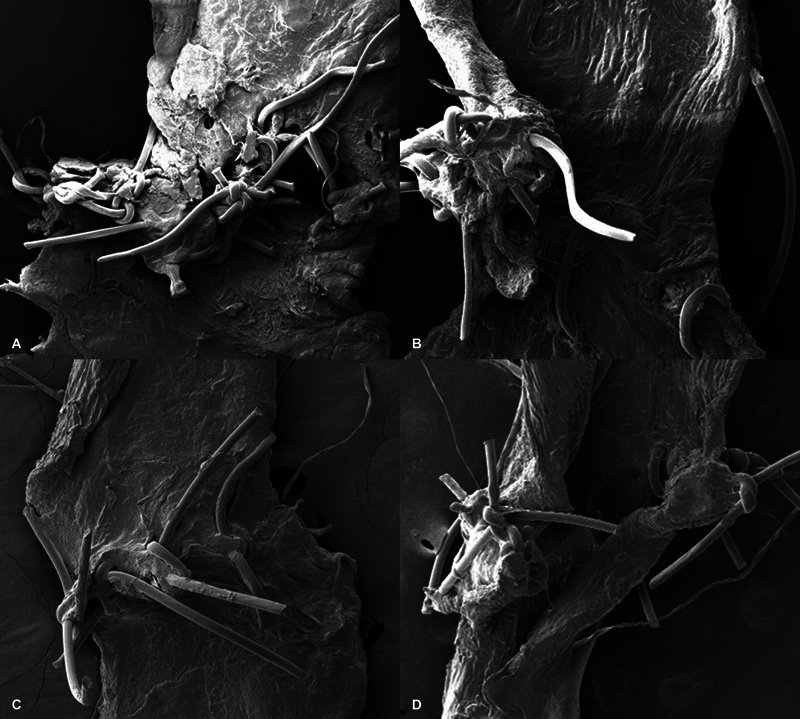
SEM images showing the status of anastomosis after the eighth trial. Note the irregular distances between the sutures, tears, and infolding of vessels in the handsewn samples ( **A, B** ) compared with the regular uniform stitches from the robot-sewn samples ( **C, D** ). SEM, scanning electron microscopy.

The vessels sutured by hand showed uneven spacing of sutures, uneven knotting structure, frequent infolding, and microtearing of vessel walls. Furthermore, there was abundant foreign body-like attachment on the sutures and vessel wall. The vessels sutured using the robotic platform exhibited even spacing of sutures, uniform knotting structure, less foreign body, better eversion of vessels, and no microtearing ( Fig. 7 ).

## Discussion

In the analysis conducted to assess proficiency, the robotic-sewn group anastomosis time plateaued from the fifth trial with an average of 2,088 ± 13.89 seconds (34.8 minutes) taking significantly longer than the handsewn group of 1320 ± 14.33 seconds (22 minutes). The biggest gap when looking at each segment of the suturing process was the time needed to cut the suture. This is due to the limit in the range of motion of the robotic arms. As for the handsewn group, proficiency was reached from the second trial. Using the robot, an earlier plateau from the fourth trial was noted for the artery and the vein from the sixth trial. The anastomosed arteries using the robot did not have severe leakage after the fifth trial and the vein after the seventh trial. Thus, an average of four to six trials may be required for proficiency and to achieve leakage-free anastomosis for arteries and an average of five to seven trials for the vein suggesting an overall learning curve of minimal four to seven trials to achieve proficiency without leakage. Taking into the fact that two vessels were used for each trial, a total of 8 to 12 vessels are required to achieve a reliable skill. Despite the arteries with an average of 0.65 ± 0.04 mm diameter, the thicker walls and stable structure made it easier to handle and reached earlier proficiency compared with the vein with a larger 0.99 ± 0.03 mm diameter. 33 These data suggest that candidates with less than 5 years of microsurgical experience could go through a learning curve that involves a minimum of four trials to reach a consistent and sufficient anastomosis of vessels. The trend of the learning curve was similar to other preclinical and clinical reports. 20 23 34 35

Looking at the number of stitches made per vessel, the robot made significantly more in the artery (7.86 ± 0.51 vs. 5.86 ± 0.67, *p* = 0.15) and also for the vein (12.63 ± 0.49 vs. 9.57 ± 0.99). This reflects the ability of the robot that allows more stitches by higher accuracy and increased dexterity. Although more stitches do not translate to higher accuracy, when the surgeon is faced with small veins and lymphatics with fragile wall, this enhanced ability to manipulate and perform fine stitches can be a deciding factor for success. To access the efficiency of the robot, the data after reaching proficiency were evaluated separately. Evaluating the time required per stitch on the seventh and eighth trials, there was no difference between the two groups suggesting the robot efficiency is equal compared with the handsewn group but required more total time due to increased number of stitches.

The SARMS score compared across each trials showed a steady increase in both groups. The first ( *p* = 0.036), second ( *p* = 0.008), and third ( *p* = 0.016) trials showed a statistically significant difference in the average scores between the two groups, but from the fourth trial onward, there was no significant difference in scores. In addition, there was no difference in patency after the fourth trial indicating similar efficacy.

The SEM findings, although descriptive in nature, were remarkably different even though vessel patency at 30 minutes was similar for both groups. Samples taken on the final trial where proficiency was reached for both groups, the vessels sutured by hand showed uneven spacing of sutures, uneven knotting structure, frequent infolding, and microtearing of vessel walls. Furthermore, there was abundant foreign body-like attachment on the sutures and vessel wall. The vessels sutured using the robotic platform exhibited even spacing of sutures, uniform knotting structure, less foreign body, better eversion of vessels, and no microtearing. These SEM finding further supports the histological evidence for precision from other studies. 27 34 This is most likely from the innate character of the robot where tremors eliminated by using a scaling system to extend the range of motion up to 20 times. 36 Especially during vessel flipping or passing the needle through the vessel, it enables much more precise handling, reducing intima damage, which ultimately can decrease the risk of thrombosis and contribute to improved overall surgical outcomes. 37 38 This is the basis of the assumption that unexpected and unknown flap failure of 3 to 5% can finally be improved.

Through this research, robot can be proficient and accurate for vessels of more or less a millimeter. However, in the realm of supermicrosurgery, the usual vessel diameter can be from 0.3 to 0.5 mm, especially in lymphatic surgery. The walls of lymphatics are very thin and fragile like veins making it very difficult to handle often leading to unsuccessful anastomosis. This is one of the reasons why there are only limited numbers of microsurgeons performing lymphovenous anastomosis, despite the high demand for surgery. The next phase of the trial needs to focus on small vessels, especially veins. In this research, the veins were average of about 1 mm and the arteries with 0.6 mm but with good definition of vessel walls. Based on the findings from this experiment, the ultimate value of the robotic anastomosis will most likely be significant over handsewn approach in these smaller caliber vessels.

There are limitations to this research. The total number of trials were limited to 8. Although a plateau is reached after the fifth trial, it cannot be conclusive on how much more improvement can be made regarding the handling of the robot. The surgeons all had up to 5 years of some microsurgery experience and the data do not reflect the novice or that of highly experienced microsurgeon. Furthermore, data are needed to evaluate the physician/students who have never been exposed to microsurgery to demonstrate the true nature of intuitiveness against data obtained from expert microsurgeons. Another shortcoming of the results is longer follow-up. For example, flap success is not determined in the first 30 minutes of microsurgery in which this experiment showed 100% patency after the second trial. Further studies are needed to reflect the outcome on flap survival using the robot.

## Conclusion

Although this is a preclinical study early in the era of robotic microsurgery platform, it can be concluded that similar patency, accuracy, and proficiency can be reached through a fast but steep learning process within four trials (anastomosis of eight vessels) compared with the classical handsewn microsurgery. The robot may take longer time compared with the handsewn anastomosis, but after proficiency is reached, this is due to the increased number of sutures reflecting higher precision and accuracy. Furthermore, valuable insight was made through the examination of SEM demonstrating the precision and accuracy of the robot that may prevent unexpected and unwanted complications.

The biggest potential of the robotic platform is providing a constant level of quality through accuracy and precision even in the hands of the beginning microsurgeons. Furthermore, microsurgeons who are not exposed to high-volume and constant cases, now can reach a level of confidence in providing microsurgery even for small caliber vessels. This platform will likely contribute to expanding the base of microsurgery and broadening the domain of microsurgeons.
